# Neglected Australian Arboviruses and Undifferentiated Febrile Illness: Addressing Public Health Challenges Arising From the ‘Developing Northern Australia’ Government Policy

**DOI:** 10.3389/fmicb.2017.02150

**Published:** 2017-11-07

**Authors:** Narayan Gyawali, Richard S. Bradbury, John G. Aaskov, Andrew W. Taylor-Robinson

**Affiliations:** ^1^Infectious Diseases Research Group, School of Health, Medical and Applied Sciences, Central Queensland University, Rockhampton, QLD, Australia; ^2^Institute of Health and Biomedical Innovation, Queensland University of Technology, Brisbane, QLD, Australia; ^3^Infectious Diseases Research Group, School of Health, Medical and Applied Sciences, Central Queensland University, Brisbane, QLD, Australia

**Keywords:** arbovirus, undifferentiated febrile illness, Australia, diagnostics, control, prevention

## Abstract

The Australian Government is currently promoting the development of Northern Australia, with an associated increase in the local population. Consequent to this is the public health threat posed by heightened human exposure to many previously neglected arboviruses that are indigenous to the region. This initiative to support economic activity in the tropical north of the continent is leading to the accelerated expansion of an infection-naïve human population into hitherto un-encountered ecosystems inhabited by reservoir animals and vectors for these arboviruses. Combined with an apparent rise in the number and impact of dramatic climate events, such as tropical cyclones and floods caused by torrential monsoonal rainfall, this heightens the potential for viral transmission to humans. More than 75 arboviruses have been identified in Australia, some of which are associated with human disease but for which routine tests are not available to diagnose infection. Here, we describe briefly the neglected Australian arboviruses that are most likely to emerge as significant agents of human disease in the coming decades. We also advocate the establishment of a thorough surveillance and diagnostic protocol, including developing new pan-viral rapid tests for primary care use to assist in the early diagnosis and correct treatment of affected patients. We propose that the implementation of these activities will enhance our understanding of the geographical range, prevalence, identification and control of neglected Australian arboviruses. This would minimise and limit the possibility of large-scale outbreaks with these agents as population and economic growth expands further into Australia’s tropical north.

## Introduction

The Australian Federal Government has over the past few years made significant efforts toward harnessing water resources and increasing development of trade and business infrastructure in the northern, tropical region of Australia in order to facilitate greater employment and associated population growth in this historically underinvested location. The Office of Northern Australia was established within the Department of Industry, Innovation and Science in 2009 with a view to providing policy advice and analysis on future initiatives to support the development of Northern Australia (Australian Government Department of Industry Innovation and Science, 2017). This office is responsible for assisting the Minister for Resources and Northern Australia to coordinate implementation of a wide-ranging policy paper, ‘Our North, Our Future,’ that describes plans for “opening up the remote and underpopulated north” and which was tabled in the Commonwealth Parliament in June 2015 (Australian Government, 2015). An obstacle to realising this positive vision of the future for Northern Australia is the unique and little studied healthcare threats that may emerge with increased human population and activity in what is currently a remote, scarcely populated and medically underserved region. Prominent among these public health challenges are the so-called ‘neglected’ Australian *ar*thropod-*bo*rne *viruses* (arboviruses), which have been infecting humans for decades but are not routinely tested in pathology laboratories (Gyawali et al., 2017). Many of these viruses lead to acute undifferentiated febrile illness (UFI), of which over half of the cases that occur in Australia each year go without diagnosis (Susilawati and McBride, 2014).

## Clinical Manifestations of UFI

Fever is defined as an abnormally high body temperature (>38°C), a common symptom of patients in healthcare settings. When the onset of fever is acute and no cause is found after taking a full history, physical examination or laboratory testing, it is classified medically as an acute UFI (Gyawali et al., 2017). As the acute UFI prolongs, it is classified as pyrexia of unknown origin (PUO) – an illness of more than 3 weeks duration accompanied by fever greater than 38.3°C (101°F) on several occasions, with failure to identify the aetiology of fever after at least 1 week of in-hospital investigation (Petersdorf and Beeson, 1961). However, this original definition from the early 1960s fails to include many self-limiting viral diseases and thus it was amended 30 years later (Durack and Street, 1991). The revised definition divided PUO into four categories; classical, nosocomial, neutropenic (immune deficient) and HIV-associated, and proposed a minimum of 3 days of hospitalisation or at least three separate outpatient presentations before this diagnosis could be applied. Categorising both acute UFI and PUO under the generic heading *undifferentiated febrile illness*, it is assumed that infection, malignancy and non-malignant inflammatory diseases are the major underlying causes (Mourad et al., 2003), where only 20–60% of UFI cases are attributed to infections alone (Jacoby and Swartz, 1973; Larson et al., 1982; Durack and Street, 1991; de Kleijn et al., 1997; Mourad et al., 2003).

## Aetiological Agents of UFI

The aetiological agents of UFI vary according to the geographical location and demographic profile of the patients. For instance, in post-industrial nations, self-limited viral infections, as well as infections with bacteria such as *Leptospira* spp., *Brucella* spp. and the atypical mycobacteria, are major causes of UFI, whereas in economically emerging countries UFI includes illnesses caused by *Mycobacterium tuberculosis*, systemic infections caused by *Salmonella enterica*, *Neisseria meningitidis*, *Plasmodium* spp., Dengue virus (DENV), Epstein-Barr virus, and other infectious agents (Boivin et al., 2000; Rongrungruang and Leelarasamee, 2001; Efstathiou et al., 2010). It has been estimated that 12–35% of hospitalised patients with UFI die from UFI-related complications (Vanderschueren et al., 2003), while about one-third to half of UFI cases remain undiagnosed (Buysschaert et al., 2004; Bleeker-Rovers et al., 2007; Robine et al., 2014). The magnitude of the problem is even greater in developing countries; in three different studies of inpatients admitted with UFI 61.3% in Thailand (Leelarasamee et al., 2004), 53% in Nepal (Blacksell et al., 2007) and 62% in Cambodia (Kasper et al., 2012) died from UFI-related disease. In such low-income countries, diagnoses of UFI are far more common due to the lack of laboratory resources, but even in a high-income nation such as Japan, which has adequate access to and quality of diagnostic tools, 28.9% of PUO is not diagnosed (Yamanouchi et al., 2013).

## UFI Diagnosis in Australia

There have been very few systematic studies of UFI in the Australian setting, with those that have been performed suggesting that a large proportion of UFI remains undiagnosed. A 3-year retrospective study in Western Australia between July 2000 and July 2003 identified 218 UFI cases, of which two-thirds were children (Ingarfield et al., 2007). Another retrospective study conducted in North Queensland from July 2008 to June 2011 showed 58.8% of patients as having no definitive diagnosis for their undifferentiated fever (Susilawati and McBride, 2014). Over the past few decades, advances in methods of diagnosis, particularly the development of molecular tests, have assisted greatly in reducing cases of UFI. However, UFI still remains a common diagnostic challenge for clinicians. Successful identification of the aetiological agents of UFI in Australia is necessary in order to develop appropriate diagnostic algorithms and to prioritise the availability of relevant diagnostic tests. This paper discusses the role and prevalence of neglected Australian arboviruses in causing UFI within Australia.

## Well Known Australian Arboviruses in UFI and Other Human Diseases

Arboviruses, which are defined as viruses that replicate in both vertebrate host and invertebrate vector and which are transmitted between vertebrate hosts by biting arthropods (such as mosquitoes, ticks, sandflies and midges), present a significant public health risk worldwide (Wilder-Smith et al., 2017). In Australia more than 75 arboviruses have been identified (Centers for Disease Control and Prevention, 2017). While only a few of these are known to cause disease in humans, there are limited or no data available regarding the potential human pathogenicity of most of these viruses (Russell, 2009). Ross River virus (RRV) and Barmah Forest virus (BFV) are the most studied Australian alphaviruses, each of which is known to cause debilitating, and sometimes chronic, polyarthritis with accompanying myalgia and lethargy (Tesh, 1982; Fraser, 1986; Vale et al., 1986). Routine diagnostic tools are available for both RRV and BFV in hospital medical microbiology and commercial pathology laboratories (Gyawali et al., 2017). Other well characterised arboviruses found in Australia are Murray Valley encephalitis virus (MVEV) and West Nile Kunjin strain virus (KUNV), both flaviviruses that cause encephalitis, while another flavivirus, DENV, is commonly associated with febrile illness or sometimes haemorrhagic fever (Russell, 1995).

Each year, a large number of cases of clinical disease are reported to be caused by arboviruses in Queensland and Northern Territory (**Table 1**), the vast majority from locations in the northern tropical region. By comparing the total incidence of arbovirus disease notifications per 100,000 population of each state and territory the relatively greater extent of undiagnosed notifications, and thereby the public health threat posed by such infections, in Queensland and the Northern Territory becomes apparent (**Table 2**). Over the current decade there is an increasing unspecified flavivirus disease burden in Western Australia but where over 96% of the population resides in the temperate south west region (Australian Bureau of Statistics, 2016).

**Table 1:** Number of notifications of diseases caused by arboviruses in Australia, January 1991 – July 2017.

State/Territory	Years	ACT	NSW	NT	QLD	SA	TAS	WA^∗^
	
Virus								
RRV	*1991–1999*	31	5,328	1,584	18,381	1,620	226	3,602
	*2000–2009*	85	7,534	2,267	19,361	1,895	295	6,453
	*2010–2017*	93	7,024	2,161	19,128	2,612	151	7,961
BFV	*1991–1999*	8	1,010	124	2,019	10	2	195
	*2000–2009*	29	4,393	572	7,272	391	7	949
	*2010–2017*	17	1,979	722	6,239	318	9	1,608
DENV	*1991–1999*	16	122	83	1,776	17	4	63
	*2000–2009*	63	720	314	2,775	124	16	383
	*2010–2017*	159	2,370	430	2,434	467	109	3,538
Unspecified flaviviruses	*1991–1999*	0	22	9	188	72	0	63
	*2000–2009*	1	55	0	165	0	0	0
	*2010–2017*	1	45	2	96	4	1	18
MVEV	*1991–1999*	0	0	NN	NN	NN	NN	NN
	*2000–2009*	0	1	6	3	1	0	7
	*2010–2017*	0	3	4	1	2	0	9
KUNV	*1991–1999*	0	0	0	NN	NN	NN	NN
	*2000–2009*	0	1	3	18	0	0	3
	*2010–2017*	0	2	2	3	0	0	5
**Total notifications**	152,464	503	30,609	8,283	79,859	7,533	820	24,857


*Data source: Australian Government, Department of Health, National Notifiable Disease Surveillance System. http://www9.health.gov.au/cda/source/cda-index.cfm. NN, not notified due to a lack of diagnostic tools in pathology reference laboratories; Viruses: RRV, Ross River; BFV, Barmah Forest; DENV, Dengue; MVEV, Murray Valley encephalitis; KUNV, West Nile virus Kunjin strain. States/Territories: ACT, Australian Capital Territory; NSW, New South Wales; NT, Northern Territory; QLD, Queensland; SA, South Australia; TAS, Tasmania; WA, Western Australia. ^∗^Although Western Australia includes northern tropical regions this state is excluded from analysis as the overwhelming majority of its population (>96%) resides in the temperate south west region not classified as part of Northern Australia.*

**Table 2:** Notification rates (per 100,000 Population) of diseases caused by arboviruses in Australia, January 1991 – July 2017.

State/Territory	Years	ACT	NSW	NT	QLD	SA	TAS	WA^∗^
	
Virus								
RRV	*1991–1999*	1.4	12.2	126.1	79.2	12.7	6.8	28.9
	*2000–2009*	2.5	11.09	107.7	48.5	12.18	6.0	31.77
	*2010–2017*	3.0	11.73	112.6	51	19.6	3.6	40.2
BFV	*1991–1999*	0.5	3.2	13.3	11.9	0.1	0.08	2.1
	*2000–2009*	0.8	6.4	26.8	18.14	2.4	0.14	4.5
	*2010–2017*	0.5	3.3	37.6	16.9	2.4	0.2	8.1
DENV	*1991–1999*	0.5	0.2	5.0	6.1	0.1	0.08	0.3
	*2000–2009*	1.8	1.0	15.4	6.8	0.8	0.3	1.8
	*2010–2017*	5.1	3.9	22.3	6.5	3.4	2.6	17.9
Unspecified flaviviruses	*1991–1999*	0	0.03	0.5	0.6	0.5	0	0
	*2000–2009*	0.03	0.09	0	0.4	0	0	0
	*2010–2017*	0.02	0.07	0.1	0.2	0.03	0.02	0.08
MVEV	*1991–1999*	NN	0	NN	NN	NN	NN	NN
	*2000–2009*	0	0	0.3	0	0.01	0	0.03
	*2010–2017*	0	0	0.2	0	0.01	0	0.05
KUNV	*1991–1999*	0	0	0	NN	NN	NN	NN
	*2000–2009*	0	0	0.2	0.03	0	0	0.02
	*2010–2017*	0	0	0.1	0	0	0	0.02


*Data source: Australian Government, Department of Health, National Notifiable Disease Surveillance System. http://www9.health.gov.au/cda/source/cda-index.cfm. NN, not notified due to a lack of diagnostic tools in pathology reference laboratories; Viruses: RRV, Ross River; BFV, Barmah Forest; DENV, Dengue; MVEV, Murray Valley encephalitis; KUNV, West Nile virus Kunjin strain. States/Territories: ACT, Australian Capital Territory; NSW, New South Wales; NT, Northern Territory; QLD, Queensland; SA, South Australia; TAS, Tasmania; WA, Western Australia. ^∗^Although Western Australia includes northern tropical regions this state is excluded from analysis as the overwhelming majority of its population (>96%) resides in the temperate south west region not classified as part of Northern Australia.*

## Neglected Australian Arboviruses in UFI

Over several decades, a large number of arboviruses have been identified in Australian mosquitoes, ticks and biting midges (Russell, 2009). Little is known about their transmission cycles, their pathogenicity for humans or their potential to cause epidemics. These neglected Australian arboviruses known to cause human disease include Alfuy (ALFV), Edge Hill (EHV), Gan Gan (GGV), Kokobera (KOKV), Sindbis (SINV) and Stratford (STRV) (**Table 3**) (reviewed by Gyawali et al., 2017). However, infection appears to manifest as predominantly mild symptoms and no outbreak of any has yet been identified (Australian Government Department of Health, 2017). In addition, there are many other arboviruses that have been isolated recently from arthropods in Australia (Russell, 2009; Johansen et al., 2013). Although close relatives of these viruses are known to cause human disease, for each its role in human infection requires further investigation. These arboviruses of unknown pathogenicity include two flaviviruses, Bamaga (BGV) (Colmant et al., 2016) and the recently discovered Fitzroy River (FRV) (Johansen et al., 2017), both of which are considered likely to represent human pathogens.

**Table 3:** Australian arboviruses identified by prototype number and source of initial isolation.

Virus name	Sero-reactive vertebrates	Infection/Disease	Source of initial isolation		^#^Geographical distribution by state or territory	Reference
					
			Strain/Year/Place	Isolated from		
§Alfuy	Wild birds and domestic fowl	Suspected polyarticular disease	MRM3929/1966/ Kowanyama, QLD	Birds: Pheasant coucal, *Centropus phasianinus*	NT, QLD, WA	Whitehead et al., 1968; Whelan and Weir, 1993; Broom et al., 1998; Johansen et al., 2003
§Edge Hill	Bandicoots, cattle, domestic fowl, humans, wallabies	Suspected arthralgia	C281/1961/Cairns, QLD	Mosquitoes: *Aedes* (*Ochlerotatus*) *vigilax*, *Culex annulirostris*	NSW, NT, QLD, VIC, WA	Doherty et al., 1963a; Marshall et al., 1982; van den Hurk et al., 2002; Mcdonald et al., 2010
^∗^Gan Gan	Cattle, horses, kangaroos, rats, wallabies	Polyarthralgia, fever	NB6057/1970/Nelson Bay, Gan Gan Army Camp, NSW	*Aedes* (*Ochlerotatus*) *vigilax*	NSW, NT, QLD, WA	Gard et al., 1973; Doherty, 1977; Briese et al., 2016
§Kokobera	Cattle, horses, humans, kangaroos, wallabies	Acute polyarticular illness	MRM32/1960/ Kowanyama, QLD	*Culex annulirostris*	NSW, NT, QLD, WA	Doherty et al., 1963a; van den Hurk et al., 2002; Johansen et al., 2013
§Kunjin	Cattle, domestic fowl, humans	Encephalitis, fever, lymphadenopathy, rash	MRM16/1960/ Kowanyama, QLD	*Culex annulirostris*	NSW, NT, QLD, VIC, WA	Doherty et al., 1963a; Marshall et al., 1982; Whelan and Weir, 1993; Johansen et al., 2013
§Stratford	Cattle	–	C338/1961/Cairns, QLD	*Aedes* (*Ochlerotatus*) *vigilax*	NSW, NT, QLD, SA	Doherty et al., 1963a; Whelan and Weir, 1993; Doggett et al., 2009


*CNS, Central Nervous System; NSW, New South Wales; NT, Northern Territory; QLD, Queensland; SA, South Australia; VIC, Victoria; WA, Western Australia. ^∗^*Bunyaviridae/Bunyavirus*; §*Flaviviridae/Flavivirus.*^#^Distribution based on virus isolation in local mosquitoes.*

It has been long since postulated that neglected indigenous arboviruses may be responsible for causing some cases of UFI observed in Australia (Doherty, 1974). Several factors are associated with the poor or absent diagnosis of UFIs. For example, when attending a patient the physician may not be aware of the existence of neglected pathogens, the causative agent may be novel, there may be no routine diagnostic tests available to detect them and/or the cost of any testing may not be considered warranted. Clinical infections with KUNV (Doherty et al., 1976; Phillips et al., 1992; Broom et al., 2001), EHV (Aaskov et al., 1993), GGV and KOKV (Hawkes et al., 1985; Boughton et al., 1986) can now be confirmed in specialised reference laboratories but only suspected KUNV infected cases are tested on a regular basis. The funding model for diagnostic pathology in Australia also restricts the likelihood that a doctor will request tests for infection with a little-known and ‘rare’ arbovirus, even if the said doctor is aware of its possible role in disease.

## Expanding Populations and Extreme Weather

The remote coastal fringe of northern Australia comprises Northern Queensland, the Northern Territory, and the Pilbara and Kimberley Ranges of Western Australia. This region is subject to a tropical, sometimes monsoonal, wet season in the summer from October to April each year (Australian Government, 2015). It is thus particularly well suited to maintaining arboviruses of potential public health importance and with the capacity to emerge as significant human pathogens (van den Hurk et al., 2010). Such neglected Australian arboviruses may have infected humans regularly for decades, thereby causing UFIs in some patients in this tropical north region without being recognised. Prior examples of arbovirus pathogens from the north causing wider-scale outbreaks, such as the incidences of MVEV in 2001, 2008 and 2011, and the KUNV equine outbreak of 2011 in south-eastern Australia (Frost et al., 2012), should also be considered.

Against this backdrop, the Australian Federal Government encourages increased settlement and economic activity in currently less populated areas that lie to the north of the Tropic of Capricorn, in particular in Northern Queensland and the Northern Territory, as an integral part of its ‘Developing Northern Australia’ community vision for the continent in the medium term (Australian Government, 2015). In this context, therefore, there is a pressing need to determine the true disease burden and geographical range of neglected indigenous arboviruses in this region. This may be achieved by establishing a regime of systematic surveillance and by performing the testing and diagnosis of UFI patients for evidence of recent infection with neglected arboviruses as well as other potential agents of UFI, such as the rickettsiae. Furthermore, to screen patients with undiagnosed fevers and other suspected cases of arboviral infection the development of novel diagnostic tools, including IgM antibody- and quantitative reverse transcription PCR (RT-qPCR) and microarray-based methods of detection of pan-alphaviruses and pan-flaviviruses (Palacios et al., 2007; Giry et al., 2017; Vina-Rodriguez et al., 2017), should be given a high research priority.

## Need for New, Clinically Available, Diagnostic Tests

**FIGURE 1:**
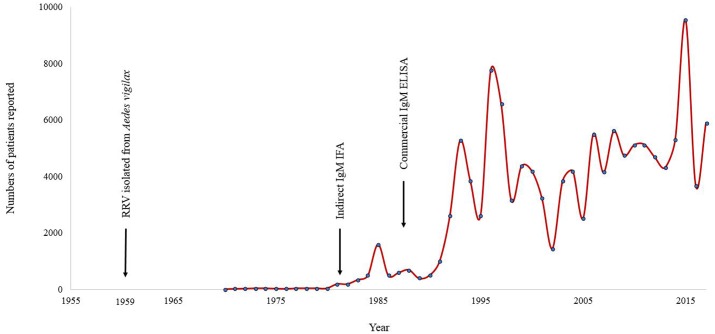
Annual number of notified cases of Ross River virus reported in Australia for the period January 1955 to July 2017. Note the increase in case identifications following the introduction of commercial diagnostic tests. ELISA, enzyme-linked immunosorbent assay; IFA, immunofluorescence assay.

Patients who presented with clinical signs and symptoms relating to RRV could not be diagnosed prior to the isolation of RRV in 1959 (Doherty et al., 1963b). Even after the identification of this virus as an agent of human disease, it took almost two decades before routine laboratory tests to diagnose infection with RRV became widely available. Following the development of an enzyme-linked immunosorbent assay (ELISA) as a commercial tool to detect anti-RRV IgM antibody (Oseni et al., 1983; Carter et al., 1985), from the early 1990s onward the number of patients diagnosed escalated dramatically to several thousand per annum (**Figure 1**). A similar experience occurred in regard to the increase in diagnosis of BFV infections and its annual notifications following the release and wide-scale availability of a diagnostic test to detect IgM against this virus (Australian Government Department of Health, 2017). Epidemic polyarthritis, the now outmoded term which was then used to describe the autoimmune conditions associated with both RRV and BFV, became a nationally notifiable disease (Hargreaves et al., 1995). These salient examples of massive growth in reported rates of infection caused by RRV and BFV compared to historical records following the introduction of commercial testing may also apply to other viral infections. Those Australian arboviruses that are currently neglected may reasonably be assumed as aetiologic in a proportion of UFI and other serious, undiagnosed infectious disease presentations in Australia.

A syndrome-based treatment protocol is in common practise at present, often used to advise the prescription of antimicrobials in empirical treatment. These pharmaceutical agents are ineffective when the UFI is caused by arboviruses; in such cases their use may add to the worsening problem of antimicrobial resistance. Early, on-site and rapid laboratory detection of neglected arboviruses could reduce the perceived need for unnecessary adoption of antimicrobials. It would also enable the early identification of outbreaks, thereby allowing time to react promptly and efficiently. Such action would limit the spread of disease, as could have been done with the recent outbreak in Latin America of the flavivirus Zika (Fauci and Morens, 2016; Lazear et al., 2016).

It is impractical to recommend multiple laboratory tests on patients for all of the neglected arboviruses. In consideration of this, we propose the prioritisation of an approach that would cover many pathogens in one test and which is applicable to a wider variety of settings. For example, we could envisage the application of a two-step protocol starting with pan-alphaviruses and pan-flaviviruses IgM antibody rapid tests, and as required followed by confirmatory detection of viral RNA by RT-qPCR.

## Potential Public Health Threat

Australian arboviral diseases, both the well-known and the relatively obscure, have a profound impact on public health and also pose a global epidemic risk as was seen previously with RRV in the Pacific Islands (Aaskov et al., 1981; Rosen et al., 1981; Tesh et al., 1981). The projected increased human activity in many areas of the tropical north of Australia will lead to fast-growing urbanisation that places immune-naïve humans into closer proximity of native reservoir wildlife, as well as of vector mosquitoes, for Australian indigenous arboviruses. Furthermore, the expansion in agriculture and other economic developments proposed for these localities will inevitably alter the ecology of the native animals and birds that act as hosts for the numerous neglected Australian arboviruses, as well as the mosquito vectors (Gyawali et al., 2017). Added to this, sudden climatic and environmental variations (Inglis, 2009), including the high rainfall, increased incidence of cyclones and resultant greater intensity of flooding associated with outbreaks of MVEV (Selvey et al., 2014) and RRV (Tall et al., 2014), have been occurring with disturbing regularity in recent years (Knutson et al., 2010), potentially bringing about an ecological shift for Australian arboviruses. Furthermore, notable close relatives of these many neglected arboviruses have already caused global pandemics in recent decades (Mayer et al., 2017).

## Conclusion and Future Directions

Vanishingly little is known of the epidemiology, transmission ecology and distribution of neglected arboviruses native to Australia. In addition, there is a dearth of information regarding the immunopathology and true disease burden, including undiagnosed UFI, which they cause. This is in the face of their potential emergence as significant human pathogens in the rapidly developing northern regions of Australia, thus posing a significant public health threat to that nation, and potentially more so globally (Gyawali et al., 2016). Further research into the highlighted areas allied to the development of diagnostic strategies and protocols, including first-line screening tests for a panel of arboviruses, would be highly beneficial to deter and potentially combat this emerging, and neglected, threat to the health of people in Northern Australia.

## Author Contributions

NG and AT-R conceived the paper and collated articles for literature review. NG prepared the tables and figure. RB, JA, and AT-R interpreted data, supervised paper writing and critically reviewed various versions of the manuscript. All authors contributed to preparation of the final version and provided consent for submission. All authors agree to be accountable for the content of the work.

## Disclaimer

RB is co-authoring this manuscript in his personal capacity and in hisrole as an adjunct academic at Central Queensland University.

## Conflict of Interest Statement

The authors declare that the research was conducted in the absence of any commercial or financial relationships that could be construed as a potential conflict of interest.
